# How dysregulation of the immune system promotes diabetes mellitus and cardiovascular risk complications

**DOI:** 10.3389/fcvm.2022.991716

**Published:** 2022-09-29

**Authors:** Diane Girard, Claire Vandiedonck

**Affiliations:** ^1^Université Paris Cité, INSERM UMR-S1151, CNRS UMR-S8253, Institut Necker Enfants Malades, IMMEDIAB Laboratory, Paris, France; ^2^Université Paris Cité, Institut Hors-Mur du Diabète, Faculté de Santé, Paris, France

**Keywords:** diabetes mellitus, atherosclerosis, inflammation, genetics, molQTL, immune cells

## Abstract

Diabetes mellitus (DM) is a chronic metabolic disorder characterized by persistent hyperglycemia due to insulin resistance or failure to produce insulin. Patients with DM develop microvascular complications that include chronic kidney disease and retinopathy, and macrovascular complications that mainly consist in an accelerated and more severe atherosclerosis compared to the general population, increasing the risk of cardiovascular (CV) events, such as stroke or myocardial infarction by 2- to 4-fold. DM is commonly associated with a low-grade chronic inflammation that is a known causal factor in its development and its complications. Moreover, it is now well-established that inflammation and immune cells play a major role in both atherosclerosis genesis and progression, as well as in CV event occurrence. In this review, after a brief presentation of DM physiopathology and its macrovascular complications, we will describe the immune system dysregulation present in patients with type 1 or type 2 diabetes and discuss its role in DM cardiovascular complications development. More specifically, we will review the metabolic changes and aberrant activation that occur in the immune cells driving the chronic inflammation through cytokine and chemokine secretion, thus promoting atherosclerosis onset and progression in a DM context. Finally, we will discuss how genetics and recent systemic approaches bring new insights into the mechanisms behind these inflammatory dysregulations and pave the way toward precision medicine.

## Introduction

Diabetes Mellitus (DM) is a worldwide major public health issue leading to increase morbidity and mortality, with over half a billion of the worldwide adult population living with DM in 2021 (International Diabetes Federation) and 1.5 million deaths due to DM complications per year according to the WHO (1). There are several forms of diabetes, type 2 diabetes (T2D) representing more than 95% of cases, combining insulin resistance and insulinopenia. Type 1 diabetes (T1D) is the second most common form of DM, representing 5–10% of all cases worldwide, and is the consequence of an auto-immune destruction of the pancreatic beta-cell. Other types of DM include gestational diabetes, and less common monogenic, atypical and syndromic diabetes (2, 3). Although the etiology is different, the common defining factor for all these different forms of DM is the hyperglycemic state clinically defined by a glycated hemoglobin (HbA1c) above ≥6.5% or a fasting plasma glucose concentration above ≥7 mmol/L (3, 4), resulting from insufficient insulin secretion that can be combined with insulin resistance. In T2D, this hyperglycemia is also frequently associated with dyslipidemia and, in the long run, both cause major organ dysfunction mainly of the heart, kidney, eyes, and lower limbs. Diabetic complications are categorized into microvascular and macrovascular. Microvascular complications group chronic kidney disease that may result in kidney failure, retinopathies that can lead to blindness and neuropathies including peripheral nerve damage leading to diabetic foot and amputation. Macrovascular complications affect big vessels such as coronary or supra-aortic arteries. Atherosclerosis is the central physiopathological mechanism of macrovascular disease, and cardiovascular disease (CVD) is a major cause of premature death in the diabetic population with cerebral ischemia (stroke) or myocardial infarction (5). Coherently, DM is a known clinical CV risk factor, as people with DM present with an accelerated and more severe form of atherosclerosis compared to the general population, significantly increasing their risk of suffering a CV ischemic event (6). Mechanisms behind this more aggressive form of atherosclerosis CVD in the diabetic population have yet to be elucidated, and are the focus of many research studies. Furthermore, although many effective tools to assess the CV risk exist in the general population, none of them are adequate to properly assess CV risk in diabetic patients (7). Thus, our incomplete understanding of DM CV complications development and progression, and the scarcity of effective tools to measure CV risk, are major issues in the treatment and prevention of diabetic CV complications. However, the chronic pro-inflammatory phenotype that diabetic patients present is a characteristic that actively participates in the onset and progression of atherosclerosis CVD (8, 9). Indeed as an inflammatory disease, atherosclerosis genesis and progression is facilitated in a pro-inflammatory setting provided by DM (10). This review aims at presenting the role of inflammation and the immune system in the development of both DM and atherosclerosis. Then we detail the known mechanisms that promote atherosclerosis onset and progression in individuals with DM. Finally, we list the common genetic regions associated to both diseases, as well as integrative omics studies that highlight variant impacting the immune system activity.

## Inflammation in diabetes mellitus and atherosclerosis

### Inflammation in T1D

T1D, formerly known as juvenile diabetes, is an auto-immune disease caused by deregulated cytotoxic T cells. These adaptive immune lymphocytes are specifically directed against pancreatic islet beta-cell epitopes, effectively destroying beta-cells and leading to insulin production deficiency. This targeted beta-cell destruction by auto-reactive T cells induces a sterile chronic inflammation called insulitis, and promotes the activation of immune B cells driving the humoral response (11). Autoantibodies, such as islet cell antibodies or anti glutamic acid decarboxylase (GAD), produced by immune B cells help for clinical diagnosis of T1D. Insulin antibodies may also be used to adapt treatments as they can indicate insulin therapy inefficiency. Although the adaptive immune system is key to T1D genesis, the innate immune system also plays an important role in the development of the disease. Indeed circulating monocytes of T1D patients have been shown to secrete interleukin 1 beta (IL-1B) and IL-6 pro-inflammatory cytokines capable of inducing Th17 pro-inflammatory cells *in vitro* (12). Once monocytes infiltrate the pancreatic tissue, they differentiate into either dendritic cells (DCs) or macrophages, both antigen-presenting cells (APCs) capable of phagocytosis and producers of pro-inflammatory cytokines. As APCs, DCs can prime naive T cells by presenting beta-cell antigens found in the pancreas to the T cells in the lymph nodes. Thus, DCs activate the adaptive immune system to attack beta-cells and promote insulitis (13, 14). Interestingly, a recent study based on single cell transcriptomics of human pancreas islets, identified ductal cells expressing MHC class II genes and a gene expression profile similar to that of tolerogenic DCs (15), consistent with a role of the exocrine pancreas in islet inflammation, as previously described in non-obese diabetic (NOD) mice (16). Chronic insulitis is further maintained by islet tissue-macrophages through the production of pro-inflammatory cytokines such as IL-1B or tumor necrosis factor alpha (TNF-a), and through their role as APC toward invading T cells (14). The neutrophil blood count decrease combined with an increase of pro-inflammatory pancreatic-residing neutrophils observed in both presymptomatic and symptomatic patients with T1D, suggest a role of neutrophils in promoting both T1D onset and progression (17). Furthermore, these pancreatic-residing neutrophils presented a pro-inflammatory phenotype and were not limited to the islets. Indeed, a 2018 study also showed that these pancreatic neutrophils produce neutrophil extracellular traps (NETs), an extracellular web-like chromatin structure known to be implicated in auto-immune disease by promoting inflammation and tissue damage (18). Similarly to neutrophils, NK cells levels in T1D are found to be decreased in the blood, which could be a consequence of an increased NK cell infiltration in the pancreas. Furthermore, NK cells of T1D patients were shown to have a higher islet cell cytotoxicity (19). Thus, several types of immune cells play a role in T1D genesis and progression (Table 1).

**Table 1:** Common immune cell function and inflammatory mechanisms between diabetes mellitus and atherosclerosis cardiovascular disease.

	**T1D**	**T2D**	**ACD**
**Inflammation type**	Chronic insulitis (11)	Chronic insulitis and systemic inflammation (8, 20–24)	Chronic systemic and plaque lesion (25)
**Role of inflammation**	Autoimmunity onset and progression Disease complications	Disease onset and progression Disease complications	Disease genesis Disease Progression Plaque instability and rupture (ischemic complications)
**Molecular mediators** Antibodies-Cytokines-Chemokines	Antibodies: used for diagnosis and treatment plan (11) IL-1B, TNF-a: maintain inflammation	IL-1B, IL-6, TNF-a, IFNg, IL-18 produced by adipose tissue: maintain a chronic inflammation that leads to insulin resistance, immune cell activation and recruitment to islets (21–23)	IL-1B, IL-6, TNF-a, IFNg, IL-18, CCL2 (MCP-1) produced by immune and endothelial cells: activation and recruitment of immune cells to the plaque (10, 25)
**Cellular mediators**			
Neutrophils	Increased pancreas infiltrates NETs extrusion in pancreas (17, 18)	NET extrusion in blood: increases with hyperglycemia (26)	Role in plaque progression: maintain inflammatory state and promote plaque instability; promote EC erosion through neutrophil traps; ROS (27, 28)
Circulating monocytes	Pro-inflammatory cytokine production: IL-1B, IL-6 Pancreas infiltration: differentiate into macrophages or DC (11, 12)	Monocyte chemoattractant production: enter AT and differentiate into macrophages Maintain chronic inflammation and insulin production defects (8, 22, 24, 29)	Atherogenesis: activated and recruited to the plaque by infiltrating the arterial wall and transform into macrophages (10)
Tissue macrophages	Islet macrophages: pro-inflammatory cytokine production (IL-1B, TNF-a, ROS) Role as APC: activate T cells (11, 14)	Adipose tissue macrophages: pro-inflammatory cytokine production (TNF-a) Islet macrophages: maintain insulitis (8, 22, 24, 29, 30)	Phagocytosis of oxLDL by macrophages that eventually transform into foam cells (10)
T-Lymphocytes	Auto-reactive T cells directed against beta-cells: lead to beta-cell destruction (11)	Insulin resistance (29, 31)	Role in plaque progression: maintain inflammatory state by producing chemokines and activating B cells and macrophages (32–36)
B-Lymphocytes	Drive the humoral response: antibody production (ICA, IAA, GAD65…) (11)	Beta-cell destruction (8, 24)	Role in plaque progression: antibody production (37)
Dendritic cells	Role as APC: autoreactive T cell priming (13, 14)	Autoreactive T cell priming (8, 22, 24, 29)	T cell priming (38)

### Inflammation in T2D

T2D is the combination of an insulin production deficiency and an insulin resistance primarily in muscle, liver and adipose tissues, the main targets of insulin. Insulin resistance is due to a disruption of the intracellular insulin signaling pathway which results in an impaired intracellular glucose uptake. Initially this decrease of insulin-stimulated glucose uptake is compensated by a beta-cell mass increase and enhanced insulin secretion. However, when the increased insulin production fails to compensate for the insulin resistance, hyperglycemia ensues, leading to T2D (39). Although the specific mechanisms behind insulin resistance are not fully understood, it has been shown that pro-inflammatory signals and inflammation, such as TNF-a, IL-1B, inhibitor of nuclear factor kappa B kinase subunit beta (IKBKB commonly known as IKKbeta), c-Jun N-terminal kinase (JNK), and NLPR3 inflammasome, are capable of disrupting insulin signaling in adipocytes or muscle cells (20). Indeed the IKKbeta/JNK signaling pathway causes a loss of insulin sensitivity, in part through an inhibiting phosphorylation by JNK of insulin receptor substrate (IRS) 1 and 2, while simultaneously activating pro-inflammatory transcription factors such as nuclear factor kappa B (NF-kB) (20). Patients with obesity, an important risk factor for T2D, often present with adipose tissue (AT) inflammation. This chronic low-grade inflammation of the AT of obese patients is reflected by their production of pro-inflammatory cytokines such as IL-6, TNF-a, IL-1B, IL-8, monocyte chemoattractant protein-1 (MCP-1: also known as CCL2), and adipokines, a group of cytokines produced by adipocytes.

Adipose tissue inflammation has multiple triggers including the increased uptake of nutrients leading to adipocyte hypertrophy and endoplasmic reticulum (ER) stress, resulting in intracellular inflammatory pathway activation (21). Noticeably, adiponectin, an anti-inflammatory adipocyte-produced hormone, that protects against insulin resistance is decreased in the plasma of obese patients (40). Interestingly, adiponectin was also found to be an independent predictor of T2D development and subsequently CV risk, as high plasma adiponectin was associated with a decreased risk of suffering a CV event among a population with T2D (41). Furthermore, both adipocyte cell death due to lipid overload and hypoxia induced by the rapid growth of AT also strongly contribute to the inflammatory state in AT (22). This eventually leads to an infiltration of both innate and adaptive immune cells in the AT, mainly infiltrating monocytes that differentiate into AT macrophages (ATM), T cells, and B cells. Similarly to muscle or liver tissue resident macrophages, ATM are the most abundant immune cells in the AT and are the major drivers of insulin resistance. Indeed, the inhibition of these pro-inflammatory macrophages protects obese mice against insulin resistance (8, 30). Pro-inflammatory CD8+ T cells promote monocyte infiltration in AT, muscle and liver, and these monocytes ultimately differentiate into pro-inflammatory macrophages (29). Interestingly anti-inflammatory regulatory T cells (Tregs) are decreased in AT of obese individuals compared to lean controls (31). Furthermore, neutrophils were found to participate in insulin resistance of hepatocytes by promoting degradation of insulin receptors (26) (Table 1).

Interestingly, high blood levels of pro-inflammatory cytokines IL-6, IL-1B, and high sensitivity C-reactive protein (hsCRP), a clinical marker of inflammation, in obese patients are predictors of insulin resistance and hyperglycemia. This suggests that in addition to local inflammation, a state of systemic inflammation occurs. Furthermore, circulating cytokines and chemokines in T2D patients are associated with blood clot formation and increased endothelial tissue damage (23). Thus, both low-grade systemic and AT chronic inflammation play a crucial role in insulin resistance, T2D onset, as well as in T2D complication development.

Inflammation also plays an important role in insulin secretion deficiency in the context of T2D. Indeed the overproduction of insulin by beta-cells in an effort to compensate for the insulin resistance causes an important ER stress in beta-cells that activates the NLRP3 inflammasome pathway leading to IL-1B production. This in turn allows for pro-inflammatory macrophage islet infiltration that also produces IL-1B, further amplifying the pro-inflammatory response (8, 24) (Table 1).

Although humoral and cellular immunity and both the adaptive and the innate immune systems are integral parts of T1D and T2D development, inflammation, or immune markers are not currently used in clinical practice for DM treatment choice, whereas the most severe and deadly complication of DM, atherosclerosis, is recognized as an inflammatory disease.

### DM macrovascular complications

As described above, patients with diabetes develop an accelerated and more severe form of atherosclerosis cardiovascular disease (ACVD), with a 3–4 times higher risk of suffering a CV event compared to the general population (5, 42, 43). However, there is an important heterogeneity of the CV risk among patients with DM. For example in patients with T2D, the incidence of a major CV event is estimated at 10.5 times higher in individuals with a CAC score >400 compared to those with a zero CAC score (42). Likewise in patients with T1D, the analysis of the EURODIAB prospective complications study revealed a reduced CV risk (0.37, 95% CI [0.18–0.76]) in patients presenting with at least 4 favorable health metrics, notably including the lower tertiles of systolic blood pressure and HbA1c levels (43). In addition, patients with DM suffer from painless myocardial infarction or silent diabetic ischemic cardiomyopathy, further increasing the difficulty of diagnosis (44). Out of 22 tools used to assess CV risk on the general population, none could accurately predict the CV risk among a diabetic population (7). The current lack of effective means to identify the CVD status of DM patients hinders CV event prevention among diabetics. Thus, identifying and elaborating methods to better predict the CV risk among the diabetic population remains crucial. More focus should be applied to taking into account inflammatory markers to assess this CV risk properly, as immune cells and inflammation are key aspects of both DM and atherosclerosis as well as CV risk progression.

### Role of the immune system and inflammation in the physiopathology of atherosclerosis

Atherosclerosis starts with a pathological intimal thickening due to endothelial dysfunction and oxidized low-density lipoprotein (oxLDL) retention in the blood vessel wall. Activation of endothelial cells then leads to circulating monocyte recruitment within the endothelium intima. Phagocytosis of the oxLDL by the monocyte-derived macrophages or vascular smooth muscle cells induces foam cell formation. Overtime, the accumulation of lipids within foam cells leads to fatty streak development. Atherosclerosis progression is characterized by fatty streak growth and the formation of a necrotic core covered by a fibrous cap. As the necrotic core, composed of lipid-rich cellular debris, develops, plaque instability increases, until the plaque eventually ruptures causing thrombosis (10). Neutrophils promote this plaque instability by eroding the endothelial wall and fibrous plaque through reactive oxygen species (ROS) secretion and neutrophil traps-extrusion, when activated by pro-inflammatory signals (27, 28). Indeed the NETs, formed of chromatin components including histones, induce an endothelial cell lysis and vascular inflammation that can lead to plaque rupture. Neutrophils also act during plaque development by enhancing monocyte recruitment through chemotactic molecules and, by secreting NETs, they activate plaque macrophage production of IL-1B (27). Throughout the entire process of atherosclerosis cardiovascular disease, a state of chronic inflammation is maintained. From the initiation of plaque onset to its progression and subsequent rupture, oxLDL activates immune and endothelial cells that will produce pro-inflammatory cytokines that in turn, maintain and promote inflammation by recruiting additional immune cells (25). More specifically the production of chemoattractants such as IL-8, MCP-1 (also known as CCL2), CCL5, and CXCL16 will amplify monocyte recruitment and promote T cell plaque infiltration, mainly pro-inflammatory Th1 interferon gamma producing cells (32). Single-cell transcriptomic analysis of human atherosclerotic carotid plaques revealed a higher concentration of T cells compared to plaque macrophages (33, 34). These pro-atherogenic effector CD4+ and CD8+ T cells presented a higher expression of activation markers such as CD69, CD38, and CCR5, both at the protein and the transcript level, compared to blood T cells. Furthermore, IL-10 producing Tregs, commonly viewed as anti-atherogenic and anti-inflammatory, can differentiate into pro-atherogenic apolipoprotein B (ApoB)-reactive T cells that produce pro-inflammatory cytokines (35). This T cell mediated effector cytokine production maintains a pro-inflammatory environment inside the arterial wall by perpetuating immune cell recruitment and activation. Furthermore, the cytotoxic activity of T cells is a major driver of necrotic core growth by inducing foam cell death (36). The T lymphocytes will also activate B cells and work in synergy to increase antibody production to maintain a chronic pro-inflammatory state. B cells are also activated through the complement system and antigen binding, and subsequent transformed into antibody producing plasma cells (37). Finally DC numbers were found to be increased in the arterial wall of atherosclerotic patients compared to healthy individuals, and the concentration of DCs inside the plaque was positively correlated to plaque instability, suggesting a role in promoting fibrous plaque erosion ultimately leading to plaque rupture (38). Overall, DCs maintain inflammation and promote plaque rupture by producing pro-inflammatory cytokines and, as APCs that prime naive T cells, by activating the adaptive immune system. The roles of these different immune cells are summarized in Table 1.

Thus, immune cells play a crucial role in atherosclerotic plaque genesis, progression, instability, and ultimately its rupture. Furthermore, therapies targeting the immune system independently of the lipid status, such as anti-inflammatory treatments, have proven effective in decreasing the CV risk in patients with atherosclerosis.

### Anti-inflammatory therapies in atherosclerosis treatments

As atherosclerosis is a chronic inflammatory disease, treatments targeting inflammation are at the forefront of research. To date, two phase III randomized clinical trials have shown beneficial effects of targeting inflammation on CV risk lowering. The CANTOS (Canakinumab Anti-inflammatory Thrombosis Outcomes Study) trial was the first to demonstrate the effectiveness of targeting inflammation to prevent CV events, with canakinumab, a monoclonal antibody directed against pro-inflammatory IL-1B cytokine, in patients with blood inflammation (hsCRP level higher than 2 mg) and previous myocardial infarction (45). This treatment led to a decrease of 15% of cardiovascular events at a median follow-up of 3.7 years, associated with a decrease of 40% of inflammation, assessed by high sensitivity C reactive protein (CRP) blood levels. Contrary to rosuvastatin, a primary prevention treatment that showed a 65% reduction in vascular events by modulating both lipid status and inflammation (46), the effect on inflammation and subsequent effect on CV risk of canakinumab was independent of lipid status. As the IL-1 pathway is a promising target for anti-inflammatory therapy, other antibodies are being developed such as anakinra, an antibody inhibiting IL-1 receptor, that has shown promising results in phase II clinical trials in both stable (47) and unstable patients (48) at high CV risk, reflecting the importance of targeting inflammation during both the acute and chronic phases of coronary artery disease (CAD).

The second randomized clinical trial involved colchicine, a known anti-inflammatory natural molecule that inhibits cytoskeleton microtubule formation, was used in the LoDoCo2 phase III clinical trial to treat patients with advanced CAD (49). There was a 31% decrease in a composite primary endpoint including CV death, myocardial infarction, ischemic stroke, or ischemia-driven coronary revascularization, in patients receiving colchicine compared to those receiving a placebo. These results are coherent with two phase II clinical trials also using colchicine for patients with either stable CAD (50), or as secondary prevention for patients having suffered a myocardial infarction (51).

Although phase III clinical trials that target cytokines, the main effectors of inflammation, have yet to be developed, two phase II clinical trials targeting IL-6 cytokine, a key pro-inflammatory effector downstream of IL-1 and TNF-a, have shown promising results. More specifically, tocilizumab, a humanized monoclonal antibody against the IL-6 receptor, decreased inflammation markers and increased myocardial salvage after myocardial infarction (52, 53).

Although atherosclerosis is defined as an inflammatory disease, and therapies targeting the immune system are undergoing clinical trial phases, DM is still considered as a metabolic disorder first, and the role of inflammation is not taken into account either for the diagnosis or the current treatments of the disease. Even so, as inflammation and immune cells are key players in the development of both T1D and T2D, clinical trials to prevent ischemic CV events in diabetic patients by targeting inflammation are underway.

### Targeting inflammation in diabetes treatment

Currently no therapy solely targeting the immune system is used to treat either T1D or T2D, however multiple studies have tested the capacity to treat or hinder DM progression using anti-inflammatory drugs.

For T1D, studies aiming to modulate the immune response in an attempt to protect beta-cell survival have had varying degrees of success (54). In 2019, a phase II randomized clinical trial with teplizumab, an anti-CD3 monoclonal antibody believed to target the auto-reactive T cells responsible for beta-cell destruction, reported a 2-year delay in T1D onset in patients at high risk for development of this clinical disease, compared to the control group given placebo (55). Another strategy modulating the T cell response through plasmid DNA vaccination showed promising results. Indeed pro-diabetic CD8+ T cells were decreased, whereas C-peptide blood levels, a by-product of insulin production, was improved in T1D individuals who were administered a DNA plasmid encoding pro-insulin, compared to control (56). Targeting pro-inflammatory molecules presented very encouraging results in a double-blind trial in which children recently diagnosed with T1D were given etanercept, a TNF antagonist. After 24 weeks of treatment, insulin dose decreased by 18% in the intervention, compared to a 23% increase in the placebo group, parallel to an increase in the endogenous production of insulin (C-peptide measurement increase of 39%, vs. a decrease by 20% in the placebo group) (57). Although targeting IL-1B showed impressive results in CV risk prevention for canakinumab and anakinra, these same molecules showed very little improvement for T1D patients (58). A major limitation of these immunomodulatory treatments are their off-target effects, limiting their distribution in individuals with T1D who can be treated with insulin.

Interestingly, treatments for T2D already in place such as insulin, metformin, glucagon-like protein peptide-1 (GLP-1) receptor agonist and sodium-dependent glucose co-transporter SGLT-2 inhibitors have been known to have pleiotropic effects including anti-inflammatory consequences. Indeed insulin has been shown to reduce pro-inflammatory pathway activity in peripheral blood mononuclear cells (PBMCs) by downregulating the expression of NF-kB pro-inflammatory transcription factor, and by decreasing the MCP-1 expression in endothelial cells (59). GLP-1, an incretin that increases postprandial insulin secretion by beta-cells, and the GLP-1 receptor agonists, possess intrinsic anti-inflammatory properties. More specifically, these hormones downregulated the expression of pro-inflammatory genes in cultured human islets, and increased Treg expression as well as TGF-beta anti-inflammatory cytokines in diabetic mice (60). SGLT-2 inhibitors, which decrease glucose blood levels by inhibiting glucose reabsorption in kidney have also intrinsic anti-inflammatory properties although the exact mechanisms behind this immunomodulation remain unknown. However, it has been suggested that SGLT-2 inhibitors may promote anti-inflammatory macrophages and IL-10 production (61). Interestingly both the incretin mimetics (GLP-1 receptor agonists) and SGLT-2 inhibitors are considered the standard of care in preventing CV complications among T2D, due to their beneficial effects on reducing CV risk (62). Finally salsalate, a non-acetylated salicylate, has anti-inflammatory properties by inhibiting NF-kB activity, and was the first blood glucose lowering drug (63).

Treatments that exclusively target inflammation are not yet established though multiple trials have tested this hypothesis with varying degrees of success. TNF-a antagonists trials have had only limited results on either glycemic control, HbA1c blood levels, insulin secretion and or sensitivity, probably due to small trial size and the short time period of treatment (9, 64). Studies targeting the IL-1 pathway using canakinumab, although effective in lowering inflammation markers as described above, have not shown any decrease of HbA1c, glucose or insulin blood levels. In the CANTOS trial, this could be at least partially explained by the recruitment selection, as the study was performed on a cohort of patients having already suffered a CV event and thus even the non-diabetic individuals had a high CV risk (65). Similarly, studies using anakinra have had modest success on insulin secretion or sensitivity but no significant effect on HbA1c. Interestingly however, IL-1B inhibition effects and trends, although not always statistically significant, were long-lasting and maintained overtime. It is important to note that none of these immunomodulating therapies aim to recover the lost beta-cells or to repair the damaged islets, but rather attempt to hinder disease progression and limit further damages.

## Hematopoiesis: Myelopoiesis and immune cell production

It has been suggested that T1D or T2D patients present with a modulated hematopoiesis (62). Whether it is the production of beta-cell targeting auto-reactive T cell production, or an increased level of circulating leukocytes mainly neutrophils and blood monocytes, these increases in key immune players of atherosclerosis development may be linked to the development of DM CVD complications (66).

### Increased myelopoiesis

**Figure 1:**
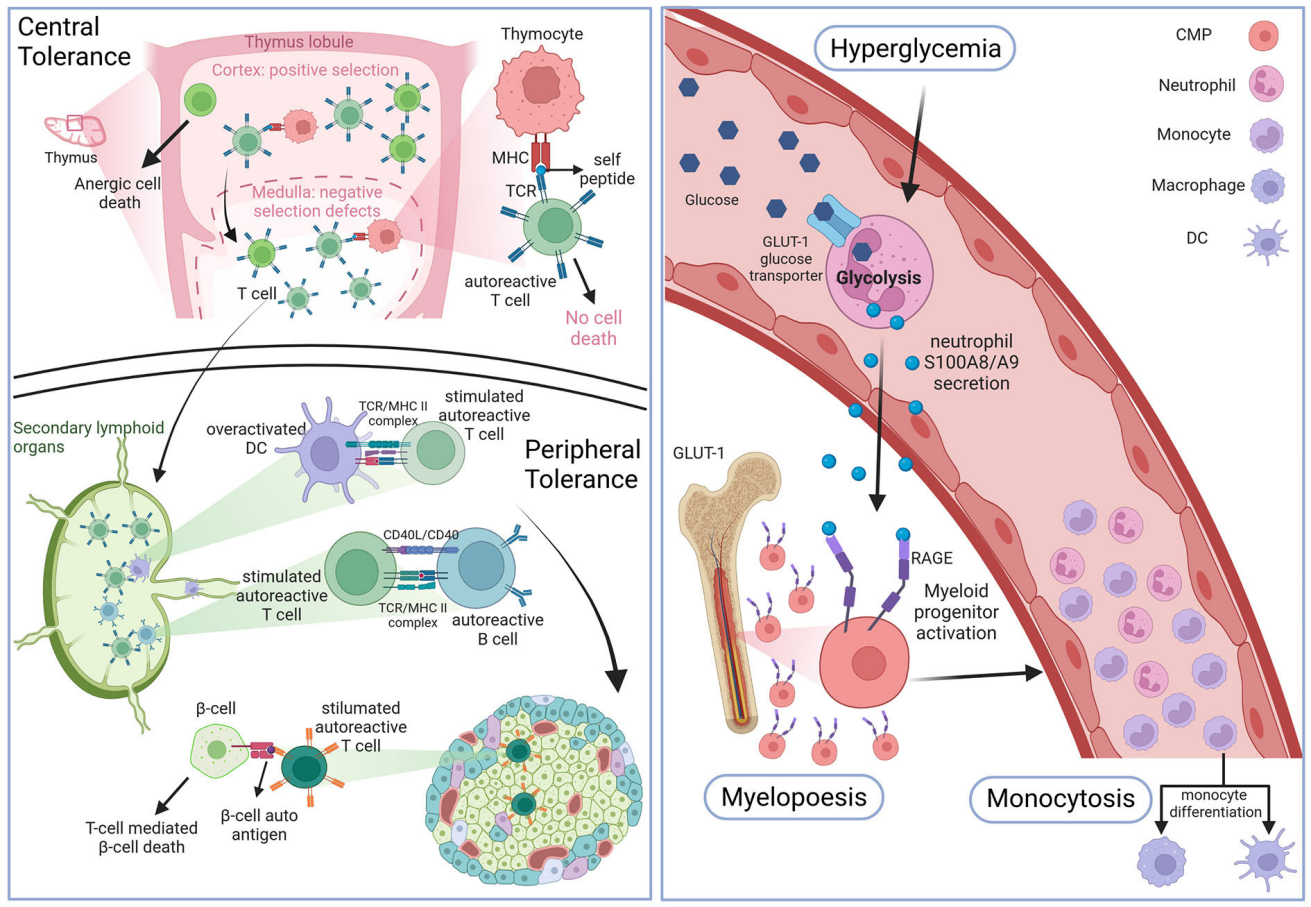
Hematopoiesis in DM. Top left panel: defects in the negative selection step of the thymic central tolerance process leads to the production of naive autoreactive T cells. Bottom left panel: antigen presenting cells (APCs) prime the naive autoreactive T cells with beta-cell and insulin autoantigens. Once activated the autoreactive T cells destroy pancreatic islet beta-cells. Right panel: high blood glucose levels enter circulating neutrophils through insulin-independent glucose transporter 1, which activates glycolysis and leads to damage associated-molecular patterns DAMPs S100A8/A9 secretion. These DAMPs will bind to receptors of advanced glycation end products (RAGE) at the surface of cellular myeloid progenitors (CMP) to promote myelopoiesis which leads to enhanced monocyte blood levels (monocytosis). Figure created with BioRender.com online tool.

The effect of hyperglycemia on immune cell production and inflammation has long been debated. However, in the last 10 years, studies have identified an indirect link between myelopoiesis, monocytosis and chronic as well as intermittent hyperglycemia. In 2013 Nagareddy et al. (67) demonstrated that chronic hyperglycemia, as it can be observed in T1D, can indirectly promote a higher myelopoiesis causing monocytosis (Figure 1). Due to an increased production by neutrophils of damage-associated molecular patterns (DAMPs) S100A8/S100A9 that bind to receptors for advanced glycation endproducts (RAGE) expressed at the surface of myeloid progenitors, myelopoiesis is enhanced, leading to an increased production of circulating monocytes (or monocytosis) (Figure 1). These results were coherent with high levels of S100A8/S100A9 measured in T1D patients at high CV risk due to bad glycemic control. As there seems to be no significant decrease in the CV risk of T2D even under good glycemic control, for a long time it was believed that hyperglycemia was linked to atherosclerosis in T1D mainly, but had only a modest effect on T2D CV complications prevention. However, since 2015, multiple studies described T2D patients with good overall glycemic control (no chronic hyperglycemia) that presented transient levels of hyperglycemia mainly after eating (68, 69). In 2020, Flynn et al. (70) successfully demonstrated that exposure to transient high levels of blood glucose could activate glycolysis in neutrophils leading to the production of S100A8/S100A9 that promote myelopoiesis and subsequent monocytosis by binding to the RAGE at the surface of myeloid progenitors (Figure 1). It should be noted that the effect of both transient and chronic hyperglycemia on myelopoiesis, mediated by neutrophil-secreted S100A8/S100A9, was independent of AGE production (67, 70). As these transient intermittent peaks of hyperglycemia were sufficient to induce an accelerated atherosclerosis development and hindered plaque regression, independently of overall good glycemic control, reflected by an absence of chronic hyperglycemia, this study may explain the differing results between T1D and T2D when it comes to lowering CV risk by controlling the HbA1c levels. Coherently with this diabetes associated monocytosis, atherosclerotic lesions present with an increased number of macrophages compared to lesions from non-diabetic individuals (71). Thus, the enhanced myelopoiesis in DM patients causing increased availability of key players of atherosclerosis onset and progression such as circulating monocytes, promotes diabetic CV complication development (66).

### Modulation of T cell differentiation

The hallmark of T1D genesis is the immune cell infiltration of pancreatic islets leading to beta-cell destruction by auto-reactive T cells mainly, as well as macrophages and B lymphocytes. It has been shown that this initial emergence of auto-reactive T cells is in part due to a default in both central and peripheral immune tolerance checkpoints (72). Studies on NOD mice were the first to show that negative selection, a thymic process during which auto-aggressive T cells are eliminated to avoid auto-immunity, seems to be defective in the context of T1D (72–74). As auto-reactive T cells are maintained, which could be due to the lack of auto-antigens presented by thymocytes including insulin itself, they migrate to the lymph nodes where they encounter APCs like DCs that prime them by presenting them with beta-cell epitopes (Figure 1). The most frequent T lymphocytes found in the pancreas of T1D individuals were cytotoxic CD8+ T cells first and CD4+ second that react to several epitopes of islet-expressed autoantigens (75). Both these types of cytotoxic T lymphocytes (CTL) were found to have a pro-inflammatory profile and were also found in the peripheral blood of T1D patients and not just in pancreatic infiltrates. Interestingly both CD8+ and CD4+ T cells can also be found in atherosclerotic plaques where they maintain the chronic inflammation state all along atherosclerosis progression, as described above. Thus, a pathology that increases the blood levels of circulating pro-inflammatory T cells may promote a faster atherosclerosis development. Regulatory T cells are known to play a role in preventing auto-immunity by resolving inflammation and secreting anti-inflammatory IL-10. Coherently with this finding, patients unable to produce Tregs develop auto-immune diseases such as diabetes (76). It is important to note, that although T2D is not an auto-immune disease like T1D, T cells also actively participate in disease development, as indicated by the presence of cytotoxic and effector T cells infiltrates in the adipose tissue that contribute to insulin resistance and systemic inflammation (39, 77).

## Modulation of immune cell activity

### Increased chemotaxis: Endothelial dysfunction

**Figure 2:**
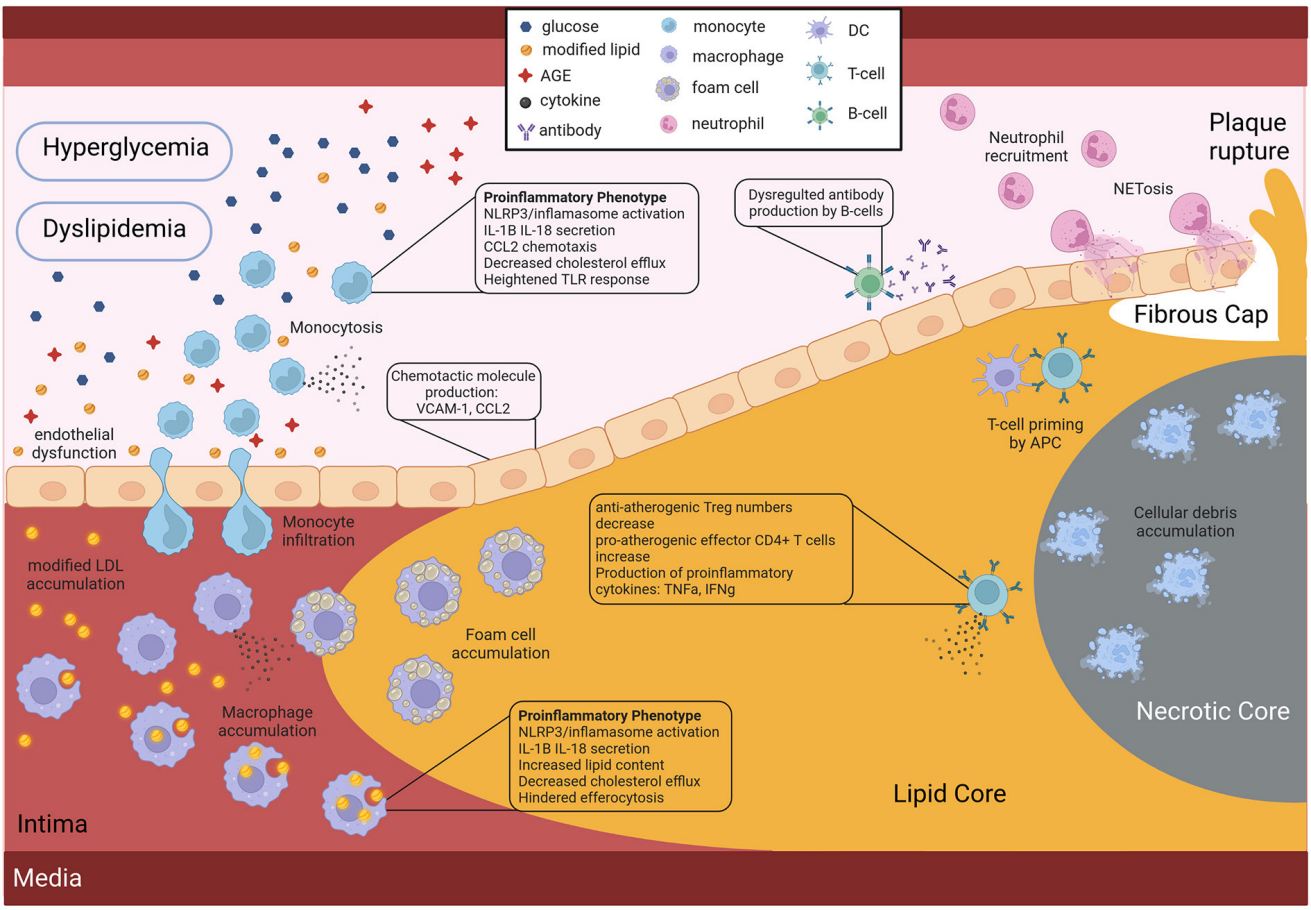
Role of the diabetic environment in promoting faster and more severe atherosclerosis CV development. Hyperglycemia and dyslipidemia, two key features of DM affect the activity of both endothelial and immune cells, by inducing pro-atherogenic phenotypes. Figure created with BioRender.com online tool.

It is now well-established that hyperglycemia leads to the development of important DM characteristics such as blood level increase of advanced glycation end products (AGEs) and reactive oxygen species (ROS) cellular production, known causes of endothelial cell dysfunction. This blood vessel wall damage is mainly reflected by a higher expression of both chemoattractants and binding proteins like vascular cell adhesion molecule 1 VCAM1, MCP-1, and IL-8 that allow for monocyte recruitment into the arterial wall, an important initial step in atherosclerosis onset (78) (Figure 2). Endothelial cells also express vascular adhesion protein-1 (VAP-1), an enzyme implicated in leukocyte extravasation as well as aldehyde, hydrogen peroxide and ammonium blood levels due to its catalytic activity in its secreted form. Interestingly, VAP-1 has been associated with inflammatory diseases including atherosclerosis (79), and serum VAP-1 levels correlated with CV risk factors (80). Furthermore, serum levels have been linked to insulin resistance (81) and adipocytes have also been shown to release VAP-1 (82). The serum level of this protein was also found to be predictive of CV events among a Taiwanese population with T2D (83). Surprisingly, however, no CV risk scores out of the 22 tested by Dziopa et al. take into account VAP-1 serum levels (7). Leukocytes such as T cells and neutrophils are also recruited at the atheroma plaque mainly through the expression of CXCR2/CCR2. Moreover, when the immune cells recruited at the lesion site encounter AGEs and ROS, they themselves start secreting pro-inflammatory cytokines such as IL-1B and IL-18. Furthermore, the eroded endothelial wall facilitates the passage of modified LDL into the blood vessel intima, and subsequent accumulation, which is the very first step of atherosclerosis genesis. Interestingly diabetic patients present with dyslipidemia and more specifically apolipoprotein B and small density (sd)LDL, as well as LDL with higher retention in atheromatous plaques, and higher production of pro-atherogenic VLDL compared to controls (84, 85) (Figure 2).

### Immune cell aberrant and uncontrolled activation

#### Monocyte pro-inflammatory phenotype acquisition

Many studies have shown that in the context of DM, monocytes present with a distinct pro-inflammatory phenotype. Indeed in 2007, a team revealed that circulating monocytes of T2D patients presented a pro-inflammatory phenotype as reflected by the production of pro-inflammatory cytokines such as TNF-a, IL-1, IL-6, and IL-8 compared to healthy controls (86) (Figure 2). Later, in 2009, Bradshaw et al. reported that blood monocytes of T1D patients spontaneously secreted pro-inflammatory IL-1B and IL-6 cytokines that promoted a pro-inflammatory Th17 cell induction, effectively promoting inflammation (12) (Figure 2). Furthermore, these pro-inflammatory monocytes in the context of DM are suggested to have a stronger response to lipopolysaccharide (LPS) or interferon (IFN)-gamma stimulation, due to an increased expression of toll-like receptors at their surface in individuals with T1D (87) and T2D (88) (Figure 2).

NLRP3 inflammasome overactivation which leads to an increased production of pro-inflammatory IL-1B and IL-18 cytokines has been reported in monocytes and monocyte-derived macrophages of diabetic individuals compared to healthy individuals by multiple studies (89–92) (Figure 2). Although the triggers have not yet been identified, it is suggested that intracellular cholesterol accumulation could be involved in this NLRP3 inflammasome overstimulation. Interestingly, it has been shown that monocytes of diabetic patients have a downregulated expression of cholesterol transporters such as ABCG1 and ABCA1, effectively inhibiting cholesterol efflux (Figure 2). This inhibition of cholesterol efflux is believed to be a consequence of the AGE blood level increase observed in diabetic patients. As mentioned above, hyperglycemia induces the production of AGEs, through excessive protein glycation, that accumulate in the circulation of diabetic patients due to their slow metabolism (93). These AGEs, when bound to their receptor (RAGE) at the surface of monocytes and macrophages, are believed to modulate gene expression by favoring a pro-inflammatory phenotype (78).

#### Macrophage activation: Pro-inflammatory phenotype acquisition

Similarly to monocytes, macrophages of both T1D and T2D individuals present a more pro-inflammatory phenotype. Furthermore, lipid loading of macrophages in the context of DM is increased (94), either due to increased very low density lipoproteins (VLDL), that are easier to uptake (95), or due to reduced cholesterol efflux (96), or a combination of both (Figure 2). This accumulation of lipid-laden macrophages as a consequence of ABCG1 downregulation in the context of T2D, was shown to promote foam cell formation (97). Finally a 2012 study further demonstrated the link between fatty acid metabolism in innate immune cells, inflammation and diabetes CV complication development. They prevented inflammatory macrophage phenotype and diabetes-accelerated atherosclerosis after deleting a fatty acid metabolism enzyme in myeloid cells (98). However, it should be noted that most studies do not demonstrate a direct link between diabetes-mediated immune cell phenotypes and atherosclerosis development, thus further studies are needed.

#### Inefficient efferocytosis

One of the reasons for the accelerated atherosclerosis development in individuals with diabetes is a faster necrotic core expansion, as revealed by the 2018 study, that used serial coronary computed tomographic angiography to measure plaque progression in T2D patients (89). This accelerated necrotic core expansion is believed to be partly due to the reduced clearance of cellular debris and apoptotic debris (Figure 2). Efferocytosis, which is the phagocytosis of dead cells, is an important function of macrophages and was shown to be impaired in T2D (Figure 2). The hindered macrophage efferocytosis is hypothesized to be the consequence of important fatty acid membrane content and dysregulated intracellular glucose uptake in a hyperglycemic environment (99, 100). Here again we have an example of how the diabetic environment affects immune cell function in a pro-atherogenic manner (Figure 2).

#### Neutrophil trap NETosis

As mentioned above NETosis markers were found to be elevated in T2D patients, and two independent studies demonstrated that hyperglycemia promotes NETs production (101, 102). Moreover, pro-inflammatory cytokine IL-6, a proven predictor of insulin resistance and T2D development, could induce NET production (101). Although the initial aim of NETs is to trap bacteria, they can be found in sterile infections including at the atherosclerotic lesion site, where they erode the endothelial wall due to the pH of the DNA, and thus destabilize the plaque (Figure 2). Thus, the increase in NETs in the diabetic population is another mechanism behind the higher risk for CV events in the diabetic population.

## Genetic and systemic approaches toward identification of individual risk

### Genetic factors contributing to T1D, T2D and atherosclerosis

**Figure 3:**
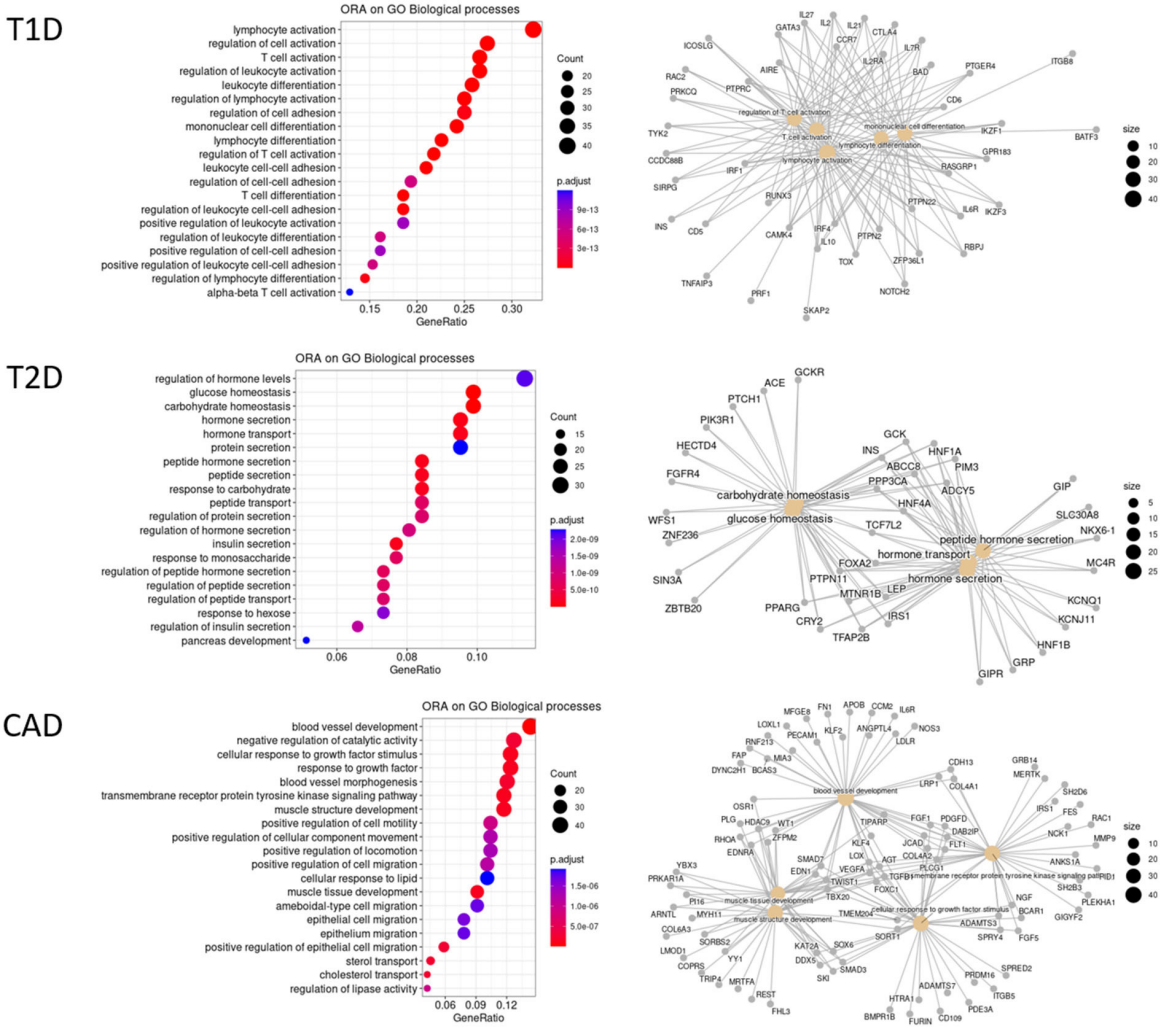
Over-representation analysis (ORA) of GWAS gene annotations for T1D, T2D, and CAD. GWAS association signals most often fall in non-coding regions. Thus, candidate genes are selected on the basis of their proximity to the GWAS lead SNPs or using integrative approaches. For some hits, several genes are considered as candidates. A total of 131 T1D candidate genes outside the MHC were collected from Onengut et al. (104), Chiou et al. (105), and Robertson et al. (106) at GWAS significant hits at a genome-wide significance. For T2D, 288 genes were considered as the most credible ones from Mahajan et al. (107) using the trans-ethnic significance threshold. The list of 347 CAD genes was collected from Erdman et al. (108) and Koyama et al. (109) at genome-wide significance threshold. HGNC official gene names were used. An ORA on these 3 datasets was conducted independently against the different GO terms and KEGG pathways with clusterProfiler R package. Only the top 20 significant enrichments at a FDR of 10% biological processes of GO are displayed here. Left panels are dotplots showing each top 20 GO term with the enrichment gene ratio (X-axis). A color code indicates the level of significance, while the circle size represents the number of genes found in the query dataset for each given GO term. Right panels are cnetplot (Gene Concept Networks) depicting the relationships between genes and GO terms.

Among etiological factors underlying these pathogenic conditions or modulating them, genetic factors play a major role. The first evidence of a genetic component in DM has been established with the Major Histocompatibility Complex (MHC) in T1D (103), as in several other autoimmune and inflammatory diseases. Since then, linkage and genome-wide association studies (GWASs), mainly led by the Type 1 Diabetes Genetics Consortium (T1DGC), have implicated tens of non-MHC genomic regions in T1D. After its last GWAS including more than 6,000 T1D patients, a total of 52 independent significant associations at genome-wide significance (i.e., 5e-8) had been reported (104). To reach more power to identify further genetic variants predisposing to pathologies, giant consortiums were constituted to conduct meta-analyses combining several GWAS datasets with massive sample sizes. Two recently published large meta-analyses more than doubled the cohort size with over 16,000 and 18,000 patients, respectively (105, 106). The former was carried out with patients from European-descent only and identified 81 significant signals including 33 new ones. The latter followed a trans-ancestry design and significant associations were found for 78 regions, 36 being new such as the *IL6R* (Interleukin 6 Receptor) locus. Altogether, with both these meta-analyses, we counted that there are currently 107 different genomic regions associated with T1D, with almost one third containing more than one independent signal. Overall, functional annotations of candidate genes in the associated regions reveal an enrichment in immune response terms (Figure 3), particularly in T cell activation signaling (e.g., *BACH2, CTLA4, PTPN2, PTPN22*) and pro-inflammatory cytokines or their receptors (e.g., *IL2, IL2RA, IL6R, IL10*, or *IL27*).

Similarly, T2D GWASs were until recently conducted in European-descent samples (110) but progressively accounted for more genetic diversity, in East-Asian samples (111). Ultimately, a huge trans-ancestral meta-analysis led by the DIAMANTE consortium, compiled 122 GWAS datasets across five ancestry groups, also including African, Hispanic and South Asian-descent samples, and recorded as much as 180,834 T2D patients and almost 1.16 million controls (107). Given the different linkage disequilibrium patterns across populations, a more stringent significance threshold was set at 5e-9. A total of 237 genomic regions with 338 distinct signals were identified. Thus, and this is a common pattern observed in most large GWAS meta-analyses for complex traits, several independent hits could be observed in 52 different regions with up to 16 for *TCF7L2* (Transcription Factor 7 Like 2), the major risk factor for T2D. As many other T2D-associated genes, *TCF7L2* is involved in blood glucose homeostasis (Figure 3). Over-representation analyses also highlight genes at play in hormone secretion. In addition, a few candidate genes are reported to be involved in inflammatory signaling pathways, either in cytokine-induced pathways (e.g., *IFNGR1, JAZF1*, or *NFE2L3*), or Tregs regulation (e.g., *MAP3K1, NLRC3*, or *PTPRJ*), or macrophage polarization (e.g., *IFNGR1, MAEA*, or *PPARG*).

Independently, variants associated with atherosclerosis have been intensively searched for using GWASs, as early as 2007 and for the past 15 years, as reviewed in Erdmann et al. (108). As of 2018, there were 163 associated regions described, among which 130 are related to 9 cellular processes linked to CAD pathophysiology, including blood pressure, lipid metabolism, diabetes or insulin resistance, transcription, mitosis or proliferation, vascular remodeling, NO signaling and relevantly inflammation with genes of the cytokine pathways (e.g., *IL5, IL6R*, or *CXCL12*) and complement components (e.g., *C1S, C2*). A recent trans-ancestry meta-analysis, on some 170,000 subjects including 26,000 cases, identified 35 additional hits (109). Taken together, there are currently 347 candidate genes for CAD, with a significant enrichment for genes involved in blood vessel or muscle development, regulation of epithelial migration or plasma lipoproteins (Figure 3).

It is worth noting that biomarkers had been associated with CAD as early as the 1960s with the correlation of blood cholesterol levels with CAD risk (Framingham Heart Study) (5). Therefore, endophenotypes contributing to atherosclerosis or to each form of DM were also considered for genetic investigation. Remarkably, most of them are quantitative traits (QTs), like BMI, lipid levels or blood phenotypes (% of cell subsets for example), amenable to genetic studies. The variants regulating them are called quantitative trait loci (QTLs). A steady effort has been made to directly map these QTLs through GWASs as they could be key players to the pathophysiology and treatments. In the case of T2D, BMI was deeply investigated with the identification of hundreds of associated variants (112). As a consequence, some T2D GWASs were BMI-adjusted (110). More than 700 regions with 1,765 variants are currently associated with blood lipid levels including LDL-C, HDL-C, TG, or total cholesterol in 7 ancestry groups (113).

Another method to investigate related traits is to perform a phenome-wide association study (PheWAS). Making use of large phenome databases such as the UKBiobank, a PheWAS tests a variant previously associated with a specific trait against all other collected traits. Thus, PheWASs have the power to connect diseases in networks based on shared genetic variants (114). A PheWAS was conducted for T2D loss-of-function variants in the Million Veteran Program cohort and revealed 3 significant associations with metabolic and inflammatory conditions (115): *ANKDD1B* (Ankyrin Repeat And Death Domain Containing 1B) was notably associated with dyslipidemia, hypercholesterolemia, blood and immune cell traits, *CCHCR1* (Coiled-Coil Alpha-Helical Rod Protein 1) with autoimmune traits, total cholesterol or NK cells, and *LPL* (Lipoprotein Lipase) with dyslipidemia or coronary atherosclerosis.

However, a key question with these multiple associations with different traits, is to determine whether the effects are independent, indicating pleiotropy, or are they related or even causative. To distinguish between these possibilities, a first method is to test for disease independence of the variant influencing the QT using a linear regression model with the disease status as covariate and an interaction term. For example, T2D variants were tested for explaining vascular traits as outcomes (115) and this analysis identified several significant interactions where the vascular outcome was modified by T2D status. To assess the potential causality of an exposure on an outcome, Mendelian randomization (MR) has become the methodology of choice (116). MR relies on the random allele distributions of genetic variants and thus uses variants associated with endophenotypes considered as exposures. Hence, it is well-established that CAD risk is positively correlated with LDL-C, and negatively correlated with HDL-C. Randomized control trials have demonstrated the beneficial effect of LDL-C lowering therapies, but none found improved outcomes with HDL-C raising drugs. Initially, MR also failed to find causation with HDL but two more recent studies were conclusive and showed that HDLs are associated with CAD independently of confounding associated phenotypes such as diabetes (117, 118).

Overall, genetic studies have uncovered myriads of variants that may impact one or several related traits. To help connecting genetic findings to pathophysiological and treatment research, an international endeavor has been undertaken to collect information, link the different studies and rank variants, notably through the GWAS catalog (https://www.ebi.ac.uk/gwas), the PheWeb tool (https://pheweb.org), or the Common Metabolic Diseases Knowledge Portal (CMDKP, https://cmdkp.org) that aggregates genetic and genomic data from several complex diseases, notably T2D, T1D, metabolic and cardiovascular diseases (119).

**Figure 4:**
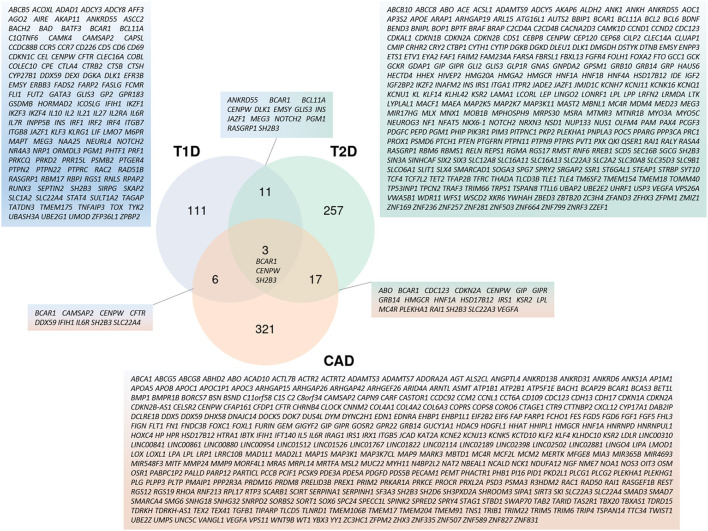
Cross-representation of candidate genes between T1D, T2D, and CAD. Venn-diagram showing number of genes specific to each disease or shared between 2 and 3 pathologies. Gene symbols for each category are listed. Genes used for this analysis are the same as the ones used in Figure 3.

Moving back to both forms of diabetes mellitus and to atherosclerosis, there is little overlap of associated regions and variants (Figure 4). Due to their different pathogenesis, it is not surprising to find poor correlation between T1D and T2D associated regions (120). If both diseases have variants affecting beta-cells, T2D variants act on beta-cell development and function, whereas T1D variants affect beta-cell function only upon immune-mediated cell perturbations (121). Remarkably, of the 5 variants that co-localized for T1D and T2D before the meta-analyses of the year 2021, only one at the *GLIS3* (GLIS family zinc finger 3) locus presented the same risk allele in both diseases while the other 4 variants displayed opposite effects (122). If we consider candidate genes neighboring GWAS-associated lead variants, 14 are in common with the two DM pathologies (Figure 4), among which only 3 are also associated with CAD: *BCAR1* (BCAR1 scaffold protein, Cas family member) whose protein product contains multiple protein-protein interaction domains and several serine and tyrosine phosphorylation sites, thus acting in different cellular pathways; *CENPW* (centromere protein W) acting in mitotic cell cycle; and *SH2B3* (SH2B adaptor protein 3) involved in growth factor and cytokine signaling.

Altogether, the variants associated with DM and CAD are frequent and have modest effects (Odds Ratios <2 except for HLA alleles in T1D), as is the case for the vast majority of variants involved in complex diseases. The impact of rare variants has been rather difficult to determine as highlighted by a well-powered study that failed to find evidence for low-frequency variants of moderate effect size in CAD (123) and likewise in T2D (124). Nonetheless, thanks to the advent of Whole-Genome-Sequencing and Whole-Exome-Sequencing approaches applied directly to patients, or indirectly to a reference panel subsequently enabling imputation of rare variants in patients, studies identified new rare variants associated with complex diseases. These had modest effects on the phenotypes but some large effects were also observed (110, 125).

Taken together, associated variants are mostly non-coding, suggesting that they rather regulate levels of gene expression. However, due to the extent of linkage disequilibrium, it is most challenging to identify the true causative variants in each associated region and also to identify which genes these variants target (126). Integrative approaches discussed below are useful to pinpoint the most likely effectors in the appropriate context.

### The utility of polygenic risk scores

Beyond population risk factors identified by GWASs, we can now compute individual risk with a polygenic risk score (PRS), a quantitative variable corresponding to the sum of allele risks at associated variants weighted by the effect size of individual risk alleles. If each of the GWAS associated variants brings little information to estimate disease risk in an individual, PRSs overcome this severe limitation and are currently of clinical value for diagnosis and treatment (127). If we focus on diseases reviewed here, the most striking PRS outcome was obtained for CAD. Indeed, PRS can predict future risk for CAD, even without family history as tested in the UKBiobank cohort where PRS provided a clear improvement to CAD prediction, with individual risks reaching 3.34 for patients in the top 5th percentile, thus equivalent to the risk conferred by monogenic forms with familial hypercholesterolemia (128). Modification of life-style and statin treatment for individuals within the PRS top 20% has proven to be beneficial with a 40–50% reduction of CV events. Thus, PRS presents a particular interest for clinical primary prevention (129). Hence PRS utility in CAD is multiple: it adds accuracy to clinical risk predictors, can help to decide who should benefit from statin prescription and facilitates estimating lifetime risk trajectories. In diabetes mellitus, T1D PRS has been beneficial for differential diagnosis between T1D and either T2D in adults (130) or monogenic forms of diabetes (131). In T1D, it has been applied to the prospective TEDDY cohort in which it could predict children at risk to have antibodies (132) and to develop the clinical disease (133). Such results give hope for primary prevention in preclinical children, knowing that secondary prevention using immunotherapy managed to delay the clinical onset (54). In T2D, PRS provides with useful information for stratification in modeling the disease risk and helps estimating lifetime risk trajectories. For example in the Million Veteran Program cohort, T2D PRS was associated with microvascular and, to a lesser extent, macrovascular T2D complications (115).

A specific portal compiling the PRS catalog across diseases has been generated (https://www.pgscatalog.org/) (134). However, caution has to be taken when using PRSs, as these scores were usually computed using GWAS summary statistics obtained in a given population. It has been demonstrated that it is crucial to match PRS with the ascendant population to determine individual risk (135). Nonetheless, the most recent trans-ethnic GWAS meta-analyses in CAD and T2D were able to generate multi-ancestry PRS more robust to population specificities (107, 109).

Altogether, PRSs have the potential to better predict disease risk and its complications, to facilitate prevention and to provide guidance in treatment decisions for precision medicine. In an effort to better define genotype-phenotype relationships, PRS can also help stratify patients. “Partitioned” or “process-specific” PRSs (pPRSs) were recently introduced to stratify risk for specific endophenotypes contributing to T1D or T2D pathogenesis, including endocrine beta-cell function or lipodystrophy (136). Such pPRSs will enhance the capacity to understand the etiological and clinical heterogeneity of the diseases as in T2D (137). A pPRS on shared variants involved in inflammation of T1D, T2D and atherosclerosis could be generated.

### Post-GWAS integrative approaches

As mentioned above, the vast majority of GWAS hits fall in the non-coding part of the genome, either in intergenic regions or in the non-coding regions of genes. This leads to the hypothesis that these variants play a regulatory role and modulate the expression levels of the genes. This modulation is known to occur through epigenomic mechanisms, including CpG methylation, chromatin remodeling and non-coding RNAs. Thus, differential methylation at 52 CpG islands in blood leukocytes was associated with higher CAD risk, two of which were causative as shown by MR (138). Similarly, a study of blood monocytes transcriptome and epigenome revealed loci associated with atherosclerosis and identified a methylation mark in the *ARID5B* (AT-Rich Interaction Domain 5B) gene. The corresponding protein is a derepressor of H3K9Me2 demethylase. Its knockdown results in a reduced expression of genes involved in atherosclerosis-related inflammation and lipid metabolism and it also inhibits phagocytosis and cell migration (139). Likewise, higher expression of *HDAC9* (histone deacetylase 9) was associated with higher concentrations of pro-inflammatory macrophages within atherosclerotic plaques, a common variant at this locus being associated with vascular calcifications and myocardial infarction risk (140). Generally, prioritization of regulatory variants in a GWAS-associated region has benefited from intersecting with epigenomic marks. In this line, T1D variants were found to be enriched in chromatin regions involved in T cell early activation (141). As for non-coding RNAs, a striking example is the highest risk variant of atherosclerosis mapping at 9p21 in a coding-gene desert region. This risk allele was shown to be associated to the linear form of the non-coding RNA *CDKN2B-AS1* (CDKN2B Antisense RNA 1, alias *ANRIL*) gene while a circular form may control rRNA maturation in vascular smooth muscle cells and macrophages and protect from atherosclerosis (142). At another level, three-dimensional chromatin architecture assayed by chromosomal capture conformation on human pancreatic islets could identify hubs of active promoters and super enhancers. Variants in these hubs were used to generate a PRS (143).

A more powerful approach is to directly test whether GWAS signals can modulate epigenomic marks considered as QTs. Variants associated to such molecular traits are known as molecular QTLs (molQTLs) (126). They usually present larger effect sizes than variants associated with the disease phenotype and therefore increase the statistical power of association studies and also facilitate the fine-mapping of the causative variant. This has been illustrated in several complex diseases, including T1D (106, 144–146), T2D (107, 110, 147), and CAD (148–150). Among molQTLs, pQTLs regulating protein expression levels raise a growing interest. If the first studies relying on quantification of protein expression by mass-spectrometry appeared somewhat disappointing, more reliable quantitative measurements are now achievable with affinity-based methods and brought positive results such as protein expression of IL-7R in T1D (151), ADIPOQ in T2D (152), or IL-1B in CAD (150). pQTLs are thus of much promising value for targeting drugs and can also be used as instruments in MR to identify causative proteins. This concept of molQTLs can be extended to cell traits, particularly in the blood. A huge study involving 563,085 European ancestry participants thus discovered 5,106 variants controlling 29 blood cell features and impacting hypercholesterolemia and hyperlipidemia by means of a PheWAS (153).

To improve our understanding of the role of molQTLs, it is critical to consider them in the right context, that is the relevant cells or tissues and the proper conditions such as the disease itself or at least disease drivers such as inflammation. The STARNET (Stockholm-Tartu Atherosclerosis Reverse Networks Engineering Task) study illustrated the importance of using the appropriate tissues in the disease context. By studying seven tissues in CAD patients, they could characterize 10 times more eQTLs than what was previously reported in healthy donors (154). Single-cell RNASeq and single-nuclei ATACSeq studies allow cell-specific resolution within tissues. Thus, an atlas of chromatin accessibility (ca) regions in coronary arteries of healthy and atherosclerotic patients was built (155). It identified 14 cellular clusters and half of the caQTLs were cell-specific. Among the top association signals for CAD, the study notably pointed to an intronic variant in the *LIPA* (lipase A) gene in a macrophage-specific chromatin accessibility element. Another study focused on single-cell transcriptome of peripheral blood mononuclear cells from 982 healthy donors. Half of the genes displayed a cell-specific eQTL. Remarkably, 19% of cis eQTL were identical to the GWAS lead SNPs in seven autoimmune diseases and MR disclosed 305 causative variants, although 60% mapped to the MHC and it is unclear how strong linkage disequilibrium in this region was accounted for (156). In a study already mentioned above, the authors complemented the meta-analysis by single nuclei ATACSeq (105). They found that the risk variants for T1D were enriched in cis-regulatory elements specific to T cells, adaptive NK cells, plasmacytoid dendritic cells, classical monocytes, and unexpectedly acinar and ductal cells of the exocrine pancreas. A novel strategy consists of integrating molQTLs into networks of co-regulation, either in a specific tissue or across multiple tissues. This has been applied in the STARNET study with the identification of 224 gene-regulatory co-expression networks that better explain the CAD heritability than the whole set of GWAS associated variants (60 vs. 22%) (157). Interestingly, cross-tissue coexpression networks were related to endocrine signaling. By MR, 218 key disease driver genes were identified at the top of these networks. They could correspond to the core genes of the omnigenic model for complex diseases (158).

Altogether, integrating genetic variants with clinical phenotypes and molecular traits from different omic layers provides with an expanded view of the mechanisms involved in pathologies and their complications and offers promising avenues toward personalized treatments.

## Conclusion

It is now well-established that inflammation plays a major role in both forms of diabetes mellitus and in atherosclerosis and that it contributes to their CV complications. So far, predictive scores of CV risk are still imperfect, particularly in the context of diabetes in which individual CV risk factors are intrinsically linked to the pathology. To help manage the patient's care and adapt the treatment targeting inflammation, it is necessary to finely characterize the inflammatory phenotypes.

Current approaches tend to classify patients according to several clinical and biological parameters with clustering approaches redefining diabetes subgroups (159). Alternative approaches envision a palette model with multidimensional continuum of multiple quantitative parameters (137, 160) in which the inflammatory component should be accounted for.

Presently, genetic variants are ill-correlated to clinical subgroups. However, focusing on specific molecular and cellular processes using integrative approaches can help dissecting the different components leading to inflammation. It could also be valuable to identify drug targets to control inflammation in a tissue specific manner. Finally, integrative approaches offer the opportunity to improve existing CV risk scores for a personalized medicine in diabetic patients.

## Author contributions

DG and CV outlined the review, collected the references, and wrote the text. All authors contributed to the article and approved the submitted version.

## Funding

DG was supported by Fondation pour la Recherche Médicale (ECO201906009041). The laboratory headed by Nicolas Venteclef was supported by ANR-11-IDEX-0005-02 Laboratory of Excellence INFLAMEX.

## Conflict of interest

The authors declare that the research was conducted in the absence of any commercial or financial relationships that could be construed as a potential conflict of interest.

## Publisher's note

All claims expressed in this article are solely those of the authors and do not necessarily represent those of their affiliated organizations, or those of the publisher, the editors and the reviewers. Any product that may be evaluated in this article, or claim that may be made by its manufacturer, is not guaranteed or endorsed by the publisher.
